# Using aggregated ethnicity categories masks inequalities in smoking prevalence in England

**DOI:** 10.1111/add.70427

**Published:** 2026-04-21

**Authors:** Eve Taylor, Harry Tattan‐Birch, Jamie Brown, Sarah Jackson

**Affiliations:** ^1^ Department of Behavioural Science and Health University College London London UK

**Keywords:** ethnicity, inequalities, prevalence, smoking, survey, tobacco

## Abstract

**Background and Aims:**

Smoking prevalence in England is usually reported using aggregated ethnicity categories, which may obscure important differences. This study aimed to: [1] estimate smoking prevalence in England for six aggregated categories; [2] examine differences between the 18 detailed constituent groups within these categories; and [3] assess whether patterns varied by gender.

**Design/Setting:**

Data were collected between 2013 and 2025 in a series of monthly cross‐sectional surveys of representative samples of the adult population in England.

**Participants:**

229 979 adults aged 18 +.

**Measurements:**

Weighted smoking prevalence was estimated for each of the 18 detailed and the six aggregated Office for National Statistics ethnicity categories. Logistic regression, adjusted for age, gender, socioeconomic status, region, survey year and survey mode examined the association between ethnicity categories and current smoking. Analyses were repeated including interactions for ethnicity by gender.

**Findings:**

Smoking prevalence in England differed substantially by ethnicity. Aggregated estimates were: Asian 12.1%, Black 11.1%, Mixed or Multiple 23.9%, White 18.0%, Arab 21.8%, and Other ethnicities 18.4%; however, these aggregates obscured important variation between detailed constituent groups. Among people from Asian ethnic groups, smoking prevalence was higher for people who were Pakistani (13.5%; adjusted odds ratio [AOR] = 1.22 [95% confidence interval (CI) = 1.07–1.39]), Bangladeshi (15.9%; AOR = 1.44 [1.25–1.71]), Chinese (11.1%; AOR = 1.25 [0.99–1.57]), and Other Asian (12.6%; AOR = 1.29 [1.07–1.55]) adults compared with people who were Indian (9.3%; reference). Among people from Black ethnic groups, smoking prevalence was higher for Black Caribbean (17.2%; AOR = 3.34, [2.87–3.89]) and Other Black (17.2%; AOR = 2.92 [2.32–3.69]) adults compared with Black African adults (7.3%; reference). Among people from Mixed or Multiple ethnicity groups, smoking prevalence was lower for White and Black African (20.5%; AOR = 0.64 [0.50–0.81]), White and Asian (20.7%; AOR = 0.70 [0.57–0.87]), and Other Mixed or Multiple ethnicity (23.4%; AOR = 0.86 [0.70–1.06]) adults compared with White and Black Caribbean adults (29.1%; reference). Among people from White ethnic groups, smoking prevalence was higher for White Irish (19.9%; AOR = 1.32 [1.18–1.48]), White Gypsy/Traveller (39.1%; AOR = 1.99 [1.43–2.76]), and Other White (25.2%; AOR = 1.19 [1.13–1.25]) adults compared with White British adults (17.5%; reference). There was little difference in interpretation between fully adjusted models and those adjusted for just survey year and survey mode.

**Conclusions:**

Smoking rates differ greatly among ethnic groups in England that are often aggregated together in research and national statistics. Such aggregation hides important inequalities, including very high smoking prevalence in certain communities.

## INTRODUCTION

An ethnicity is a group of people who identify with each other based on shared culture, heritage, religion and identity. There are health inequalities between ethnic groups in the UK, with the poorest health reported among Asian Bangladeshi and Pakistani communities and Gypsy and Traveller communities [1]. Incidence rates and mortality from cardiovascular disease are higher among Asian communities compared with the national average, and Black communities have a higher‐than‐average incidence of and mortality from hypertension and stroke [2]. Cancer is generally more prevalent among White groups; however, specific cancers are more common among minority ethnic groups, such as higher rates of prostate cancer among Black men and higher rates of mouth cancer among Asian women [2]. There are many reasons for these health inequities, such as greater deprivation among certain ethnic groups, structural racism, issues with health literacy, healthcare discrimination and behavioural risk factors such as smoking [1, 2, 3, 4, 5].

Smoking prevalence in England is often reported using six broad aggregated ethnicity categories: White; Asian/British Asian; Black/Black British; mixed or multiple ethnicities; Arab; and ‘other’ ethnicities [6, 7]. This approach may mask important differences between constituent groups. For example, Gypsy and Traveller communities, who have particularly high smoking rates, are typically combined with other White ethnicities in national reporting, which masks their elevated prevalence for smoking [8]. Similar patterns have been noted when more detailed categorisations are applied in other contexts, such as maternity services in London, where smoking prevalence among White and Black Caribbean women (12%) was substantially higher than among women in other mixed ethnic groups (0%–5.4%), and where smoking prevalence among White Irish women (9%) was higher than among women in other White groups (4.8%–5.9%) [9]. Variations in smoking rates have also been reported in Aotearoa New Zealand, depending on how Māori and Pacific Island groups are categorised in surveys [10]. Despite these examples, nationally representative reporting of smoking prevalence by detailed ethnicity categories is limited in England, highlighting the need for disaggregated analyses.

While smoking is generally more prevalent among men than women in England [6], varying cultural attitudes towards gender and smoking mean that gender differences in smoking are not likely to be uniform across ethnicities [7, 11, 12, 13].

Using data from the Smoking Toolkit study, pooled between 2013 and 2025, this study aimed to:

1. estimate smoking prevalence for six aggregated ethnicity categories in England;
2. examine differences in smoking prevalence between the 18 detailed constituent groups within each aggregate ethnicity category; and
3. assess whether these patterns varied by intersections of ethnicity and gender.

## METHODS

### Design

Data were drawn from the Smoking Toolkit Study, an ongoing monthly cross‐sectional survey of adults (aged ≥16 years) in England [14, 15]. The study uses a hybrid of random probability and simple quota sampling to select a new sample of approximately 1700 adults in England each month. A comprehensive question on ethnicity was added in January 2013. Data were originally collected via face‐to‐face interviews, but data collection switched to telephone interviews from April 2020 onwards; the two modes show good comparability on key socio‐demographic and smoking indices [16]. All participants provided oral consent.

### Participants

A total of 245 211 adult (aged ≥18 years) participants in England were included between January 2013 and May 2025. Data on ethnicity were not collected between April and August 2020, so the data for these waves were excluded (*n* = 8459), as well as participants who reported ‘do not know’ or refused to give their ethnicity (*n* = 1356). Participants who were missing data on outcome (*n* = 739) or covariate variables (*n* = 4652) were excluded. Owing to the small sample sizes (*n* = 765, 0.3%), people who identified their gender as ‘in another way’ were also excluded from all analyses, resulting in an analytical sample of *n* = 229 979.

### Measures

#### Smoking status

Smoking status was assessed by asking participants which of the following statements best applied to them: (a) I smoke cigarettes (including hand‐rolled) every day; (b) I smoke cigarettes (including hand‐rolled), but not every day; (c) I do not smoke cigarettes at all, but I do smoke tobacco of some kind (e.g. pipe, cigar or shisha); (d) I have stopped smoking completely in the last year; (e) I stopped smoking completely more than a year ago; (f) I have never been a smoker (i.e. smoked for a year or more). Responses were coded ‘current smoking’ (a–c), ‘former smoking’ (d–e) and ‘other’ (f). For the regression models, smoking status was coded as ‘current smoking’ (a–c) or ‘other’ (d–f).

#### Detailed ethnicity

Ethnicity was assessed following the Office for National Statistics (ONS) ethnicity categories recorded in the national census [17]. Participants were asked which group they considered they belonged to from the following list: (1) White—English/Welsh/Scottish/Northern Irish/British; (2) White—Irish; (3) White—Gypsy or Irish Traveller; (4) White—any other White background; (5) Mixed—White and Black Caribbean; (6) mixed—White and Black African; (7) mixed—White and Asian; (8) mixed—any other mixed or multiple ethnic background; (9) Asian/Asian British—Indian; (10) Asian/Asian British—Pakistani; (11) Asian/Asian British—Bangladeshi; (12) Asian/Asian British—Chinese; (13) Asian/Asian British—any other Asian background; (14) Black—African; (15) Black—Caribbean; (16) Black—any other Black/African/Caribbean background; (17) Arab; (18) any other ethnic group; (19) do not know (excluded); (20) refused to say (excluded).

#### Aggregated ethnicity

Aggregated ethnicity categories were defined as ‘White’ (White British, White Irish, White Gypsy/Traveller, other White ethnicity), ‘Asian, British Asian’ (Asian Indian, Asian Pakistani, Asian Bangladeshi, Asian Chinese, other Asian ethnicity), ‘Black, Black British’ (Black African, Black Caribbean, other Black ethnicity), ‘mixed or multiple ethnicities’ (White and Black Caribbean, White and Black African, White and Asian, multiple other ethnicities), ‘Arab’ or ‘other ethnicity’.

#### Covariates

Covariates included survey year (continuous), gender (man, woman), age (continuous), geographic region (North East, North West, Yorkshire Humber, East Midlands, West Midlands, East of England, London, South East, South West) and socio‐economic position (ABC1, C2DE). Socio‐economic position was indexed by a measure of occupational social grade, categorised based on National Readership Survey classifications as AB (higher and intermediate managerial, administrative and professional); C1 (supervisory, clerical and junior managerial, administrative and professional); C2 (skilled manual workers); D (semi‐skilled and unskilled manual workers); or E (state pensioners, casual and lowest grade workers, unemployed with state benefits only) [18]. In addition, we included a binary variable to account for changes in survey methodology in April 2020 from in‐person to telephone interviews, resulting from the COVID‐19 pandemic [14].

### Analysis

First, weighted smoking prevalence was estimated for each ethnicity using the aggregated (6‐level) and detailed (18‐level) categorisations. Second, logistic regressions examined the association between both ethnicity variables and current smoking, adjusted for: (1) year, survey mode; (2) year, survey mode, age, gender; and (3) year, survey mode, age, gender, geographic region, socio‐economic position. Analyses were repeated including two‐way interactions between ethnicity and gender, adjusted for age, geographic region, socio‐economic position, year and survey mode.

All analyses were weighted to match the English population profile [19]. Data analyses were not pre‐registered and should be considered exploratory.

## RESULTS

**FIGURE 1:**
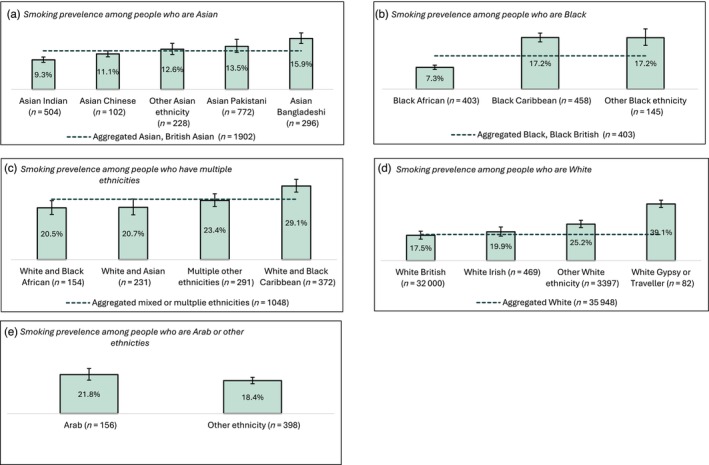
Smoking prevalence by ethnicity in England; pooled 2013–2025.

Among adults aged ≥18 years, smoking prevalence pooled between 2013 and 2025 differed substantially among ethnic groups in England (Figure 1; Table 1). The prevalence of ever smoking followed a similar pattern to current smoking; however, former smoking was only greater than current smoking among people who were White British or Irish (Figure S1).

**TABLE 1:** Smoking prevalence by ethnicity; pooled 2013–2025.

	*n*	% (95% CI)	aOR (95% CI)	*P*
Aggregated Asian, British Asian	16 970	12.1 (11.5–12.6)	–	–
Asian—Indian	5781	9.3 (8.5–10.1)	1.00	Ref.
Asian—Pakistani	6138	13.5 (12.6–14.4)	1.22 (1.07–1.39)	**0.002**
Asian—Bangladeshi	2072	15.9 (14.3–17.8)	1.44 (1.22–1.71)	**<0.001**
Asian—Chinese	1038	11.1 (9.2–13.3)	1.25 (0.99–1.57)	0.064
Other Asian ethnicity	1941	12.6 (11.0–14.3)	1.29 (1.07–1.55)	**0.007**
Aggregated Black, Black British	10 008	11.1 (10.5–11.8)	–	–
Black—African	6073	7.3 (6.6–8.0)	1.00	Ref.
Black—Caribbean	3042	17.2 (15.8–18.8)	3.34 (2.87–3.89)	**<0.001**
Other Black ethnicity	893	17.2 (14.6–20.2)	2.92 (2.32–3.69)	**<0.001**
Aggregated mixed or multiple ethnicities	4654	23.9 (22.5–25.2)	–	–
White and Black Caribbean	1341	29.1 (26.4–31.9)	1.00	Ref.
White and Black African	801	20.5 (17.5–23.8)	0.64 (0.50–0.81)	**<001**
White and Asian	1207	20.7 (18.2–23.3)	0.70 (0.57–0.87)	0.**001**
Multiple other ethnicities	1305	23.4 (21.0–26.0)	0.86 (0.70–1.06)	0.150
Aggregated White, White British	195 324	18.0 (17.9–18.2)	–	–
White—British	180 369	17.5 (17.3–17.7)	1.00	Ref.
White—Irish	2427	19.9 (18.1–21.7)	1.32 (1.18–1.48)	**<0.001**
White—Gypsy/Traveller	192	39.1 (31.9–46.7)	1.99 (1.43–2.76)	**<0.001**
Other White	12 336	25.2 (24.4–26.0)	1.19 (1.13–1.25)	**<0.001**
Arab	762	21.8 (18.7–25.2)	–	–
Other ethnicities	2261	18.4 (16.7–20.3)	–	–

*Note*: The model is adjusted for age, gender, socio‐economic position, geographic region, survey mode and survey year. aOR, 95% CI and *P*‐values reflect pairwise comparisons with the designated reference category within each aggregated ethnic group. Aggregated ethnicity was not included in the models but smoking prevalence in these categories is presented for the purpose of comparison. Regression models and percentages are weighted, *n* values are unweighted. Bold = *p* < 0.05.

Abbreviations: aOR = adjusted odds ratio; 95% CI = 95% confidence interval.

In each ethnic group, smoking was more prevalent among people from less advantaged (C2DE) social grades compared with those who were more advantaged (ABC1). Smoking status varied across regions in England; however, owing to the small sample sizes for many ethnic groups, the estimates were imprecise (Tables S1–S4).

### Smoking by aggregated ethnicity categories

When these ethnicities were aggregated, smoking prevalence was estimated to be 21.8% among people who were Arab, 12.1% among people who were Asian, 11.1% among people who were Black, 24.0% among people who had mixed or multiple ethnicities, 18.1% among people who were White and 18.5% among people who reported an ‘other’ ethnicity (Table 1).

### Smoking by detailed ethnicity categories

As there was little difference in the interpretation of models between the three levels of adjustment, fully adjusted models are therefore presented below (Table S4).

Among people who were Asian, smoking prevalence was greater among people who were Asian Pakistani (13.5%; aOR = 1.22; 95% CI = 1.07–1.39; *P* = 0.002), Asian Bangladeshi (15.9%; aOR = 1.44; 95% CI = 1.22–1.71; *P* < 0.001) or from other Asian ethnicities (12.6%), compared with people who were Asian Indian (9.3%) (Figure 1; Table 1). Smoking prevalence also appeared to be slightly higher among those who were Asian Chinese (11.1%; aOR = 1.25; 95% CI = 0.99–1.57; *P* = 0.064), although the 95% confidence interval included the possibility of non‐significance (Figure 1; Table 1). Similar differences between ethnicities were observed among men, with smoking prevalence being substantially greater among Asian Bangladeshi men (24.4%) and Asian Pakistani men (18.9%) compared with Asian Indian men (13.1%). Differences among women were less apparent, with smoking prevalence markedly lower among all of the communities of Asian women (4.2%–6.6%) compared with Asian men (13.1%–24.4%). However, the differences in smoking rates between men and women varied significantly between groups (Figure S2; Table 2).

**TABLE 2:** Predicted smoking prevalence by ethnicity and gender; pooled 2013–2025.

	Men				Women				Men vs women interaction
	*n*	% (95% CI)	aOR (95% CI)	*P*	*n*	% (95% CI)	aOR (95% CI)	*P*	
Aggregated Asian, British Asian		17.3 (16.5–18.1)	–	–		4.9 (4.4–5.5)	–	–	–
Asian—Indian	3319	13.1 (11.8–14.4)	1.00	Ref.	2462	4.2 (3.4–5.1)	1.00	Ref.	Ref.
Asian—Pakistani	3806	18.9 (17.6–20.4)	1.28 (1.10–1.49)	**0.001**	2332	4.9 (4.1–5.9)	0.92 (0.69–1.23)	0.569	**0.046**
Asian—Bangladeshi	1199	24.4 (21.8–27.2)	1.72 (1.42–2.09)	**<0.001**	873	4.3 (3.1–6.1)	0.77 (0.50–1.18)	0.228	**0.001**
Asian—Chinese	532	16.1 (13.0–19.7)	1.26 (0.95–1.67)	0.103	506	6.3 (4.4–9.0)	1.56 (1.00–2.43)	0.051	0.435
Other Asian ethnicity	1062	17.7 (15.2–20.5)	1.28 (1.02–1.59)	**0.029**	879	6.6 (5.1–8.5)	1.48 (1.04–2.11)	**0.031**	0.492
Aggregated Black, Black British		13.9 (12.8–15.0)	–	–		8.6 (7.8–9.5)	–	–	–
Black—African	3009	9.8 (8.6–11.0)	1.00	Ref.	3064	4.8 (4.0–5.7)	1.00	Ref.	Ref.
Black—Caribbean	1314	21.0 (18.7–23.6)	2.92 (2.37–3.60)	**<0.001**	1728	14.4 (12.7–16.3)	4.31 (3.41–5.45)	**<0.001**	**0.015**
Other Black ethnicity	433	21.7 (17.7–26.4)	2.75 (2.02–3.74)	**<0.001**	460	12.9 (9.9–16.6)	3.33 (2.32–4.76)	**<0.001**	0.429
Aggregated mixed or multiple ethnicities		25.2 (23.2–27.3)	–	–		22.7 (20.9–24.5)	–	–	–
White and Black Caribbean	579	31.3 (27.0–35.9)	1.00	Ref.	762	27.4 (24.1–31.0)	1.00	Ref.	Ref.
White and Black African	380	22.7 (18.3–27.8)	0.65 (0.46–0.92)	**0.016**	421	18.4 (14.6–22.9)	0.62 (0.44–0.87)	**0.006**	0.768
White and Asian	612	23.4 (19.9–27.4)	0.74 (0.55–1.00)	0.053	595	17.8 (14.7–21.4)	0.66 (0.49–0.89)	**0.006**	0.370
Multiple other ethnicities	604	22.7 (19.2–26.6)	0.72 (0.53–0.98)	**0.036**	701	24.0 (20.7–27.6)	1.01 (0.77–1.32)	0.924	0.085
Aggregated White, White British		19.4 (19.1–19.7)	–	–		16.8 (16.5–17.0)	–	–	–
White—British	90 137	18.5 (18.0–18.8)	1.00	Ref.	90 232	16.5 (16.2–16.7)	1.00	Ref.	Ref.
White—Irish	1272	22.4 (19.9–25.0)	1.40 (1.21–1.63)	**<0.001**	1155	17.4 (15.1–19.9)	1.23 (1.04–1.46)	**0.018**	0.262
White—Gypsy/Traveller	109	41.9 (32.2–52.2)	2.15 (1.40–3.31)	**0.001**	83	35.1 (25.2–46.5)	1.80 (1.08–2.99)	**0.023**	0.600
Other White	5613	30.5 (29.2–31.8)	1.41 (1.32–1.51)	**<0.001**	6723	20.6 (19.6–21.6)	0.99 (0.93–1.07)	0.862	**<0.001**
Arab	465	27.6 (23.3–32.3)	–	–	297	12.0 (8.4–16.9)	–	–	–
Other ethnicities	1189	22.7 (20.1–25.4)	–	–	1072	13.7 (11.6–16.2)	–	–	–

*Note*: The model is adjusted for age, gender, socio‐economic position, geographic region, survey mode and survey year, and includes an interaction between gender and smoking. aOR, 95% CI and *P*‐values reflect pairwise comparisons with the designated reference category within each aggregated ethnic group. Aggregated ethnicity was not included in the models but smoking prevalence in these categories is presented for the purpose of comparison. Regression models and percentages are weighted, *n* values are unweighted. Bold = *p* < 0.05.

Abbreviations: aOR = adjusted odds ratio; 95% CI = 95% confidence interval.

Among people who were Black, smoking prevalence was greater among people who were Black Caribbean (17.2%; aOR = 3.34; 95% CI = 2.87–3.89; *P* < 0.001) or from other Black ethnicities (17.2%; aOR = 2.92; 95% CI = 2.32–3.69; *P* < 0.001), compared with people who were Black African (7.3%) (Figure 1; Table 1). Similar differences between ethnicities were observed among men and women, with smoking prevalence lower among women (4.8%–14.4%) than among men (9.8%–21.7%). However, the differences in smoking rates between men and women varied significantly between groups (Figure S2; Table 2).

Among people who had mixed or multiple ethnicities, smoking prevalence was lower among people who were White and Black African (20.5%; aOR = 0.64; 95% CI = 0.50–0.81; *P* ≤ 0.001) or White and Asian (20.7%; aOR = 0.70; 95% CI = 0.57–0.87; *P* = 0.001), compared with people who were White and Black Caribbean (29.1%) (Figure 1; Table 1). Prevalence also appeared to be slightly lower among those who had multiple other ethnicities (23.4%; aOR = 0.86; 95% CI = 0.70–1.06; *P* = 0.150), although the 95% confidence interval included the possibility of no difference (Figure 1; Table 1). Similar differences between ethnicities were observed among men and women; however, the 95% confidence intervals included the possibility of no difference for some comparisons (Figure S2; Table 2).

Among people who were White, smoking prevalence was greater among people who were White Irish (19.9%; aOR = 1.32; 95% CI = 1.18–1.48; *P* < 0.001), White Gypsy or Traveller (39.1%; aOR = 1.99; 95% CI = 1.43–2.76; *P* < 0.001) or from other White ethnicities (25.2%; aOR = 1.20; 95% CI = 1.14–1.27; *P* < 0.001) compared with people who were White British (17.5%) (Figure 1; Table 1). Similar differences between ethnicities were observed among men and women; however, the 95% confidence intervals for women included the possibility of no difference (Figure S2; Table 2). The differences in smoking rates between men and women were greater for people who were from other White ethnicities compared with White British people (*P* < 0.001).

## DISCUSSION

Overall, smoking prevalence varied considerably between and within ethnic groups in England. Smoking rates were generally lower among women than men; however, the differences between genders were greater among some ethnic groups. The findings show that the reliance on aggregated ethnicity groups, as opposed to more detailed ethnicity groups, in smoking research and policy greatly overestimates or underestimates smoking rates among certain communities.

Smoking rates varied substantially among ethnic groups, particularly among the Black/Black British communities. Smoking rates were over twice as high among people from Black Caribbean (17.2%) or from other Black ethnicities (17.2%), compared with people who were from Black African (7.3%) communities. Smoking statistics are often only reported for these groups aggregated as Black/Black British (11.1%); however, this conceals substantially higher smoking rates among specific communities. Smoking rates were also substantially greater among White Gypsy Traveller groups than other White ethnicities, which has been previously reported using this data set [8]. Similar to previous research, smoking was more common among men than among women across all ethnicities [19, 20], a finding that is common across every World Health Organization (WHO) region [21]. However, this was most apparent among Asian, Black and Arab communities, compared with White communities. These differences may reflect specific cultural attitudes towards tobacco use, for example in Asian communities where smokeless tobacco use is more culturally acceptable among women than cigarette smoking [22, 23].

Smoking prevalence was greater among less advantaged socio‐economic grades compared with more advantaged grades; however, the sample sizes for some groups were too small to compare gradients precisely. The interactions among socio‐economic position, ethnicity and smoking are complex, and are underpinned by the structural racism and discrimination faced by minority ethnic groups in the UK [1, 2, 3, 4, 5]. Previous research has reported that the strong socio‐economic gradient in smoking observed among White and Asian Chinese groups was not apparent among other Asian groups, suggesting that traditional class or occupation‐based explanations for smoking patterns may not apply uniformly across different communities [24].

Ethnicity is a multi‐dimensional concept, with many different elements likely contributing towards varying smoking rates. For example, smoking rates have been found to vary by religion in the UK [25], which can be connected to culture and ethnicity. The effect of migration patterns on smoking also varies across ethnicities, as smoking prevalence is substantially higher among White first‐generation migrants from Eastern European countries, and Turkey and Greece, than among people from the second generation [24]. On the other hand, people who are second‐generation Asian Pakistani or Bangladeshi are more likely to smoke than people from these communities who are from the first generation [26]. These differences may be linked to cultural norms towards smoking and how they shift and change within communities over time.

The findings suggest that more exploration of ethnicity data is required when commissioning smoking cessation services. Over‐simplified ethnic categorisation may lead local authorities to overlook specific communities requiring additional support, thereby exacerbating health inequalities. When reporting smoking by ethnicity, we should also consider differences by gender. Patterns in smoking rates between genders are not equivalent across ethnicities, and the low levels of smoking among women in certain communities may obscure high rates of smoking among men from these communities. Greater efforts are therefore needed within the research and public health communities to collect, analyse and report detailed ethnicity data, enabling the clearer identification of health inequities and more effectively targeted strategies to reduce them.

These findings are specific to England and reflect how ethnicity is defined and shaped in this context. However, evidence from other countries also shows that smoking prevalence estimates vary depending on how ethnicity is categorised and the level of granularity used [10, 27]. International researchers should therefore carefully consider the level of granularity in the collection and reporting of ethnicity data, ensuring that classifications are appropriate to their specific national and cultural context and contribute to reducing health inequities.

Our research has several limitations. First, data were aggregated over a 12‐year period to maximise the sample size; therefore, the estimates do not reflect current smoking prevalence but the average smoking prevalence across the study period. Second, we had small subgroup sample sizes for some ethnic groups; therefore, the smoking estimates for regional analyses may be imprecise. Our sampling methods moved to telephone interviews in 2020, which may be less accessible to certain communities that have a greater proportion of people who do not speak English fluently and would likely decline to participate in a telephone survey. Also, Arab and other ethnicities represent aggregate categories; however, we were unable to present data on their constituent groups as these are not captured in our survey. Finally, people define their ethnicity in many differing ways in England, with over 250 different options used to record ethnicity by the National Health Service (NHS) [28], and therefore our 18 categories may not fully represent the many different ethnic identities in England.

In conclusion, smoking rates differ greatly between ethnic groups in England. Researchers should be cautious of reporting oversimplified aggregated ethnicity categorisation, as this may mask true smoking rates among certain communities. Policymakers and clinical commissioning groups should also be conscious of differing smoking patterns nationally and among their local communities to ensure resources are provided to the communities that need greater smoking cessation support.

## AUTHOR CONTRIBUTIONS

**Eve Taylor:** Conceptualization; writing—original draft; writing—review and editing; formal analysis; methodology. **Harry Tattan‐Birch:** Conceptualization; methodology; writing—review and editing; writing—original draft. **Jamie Brown:** Funding acquisition; conceptualization; methodology; supervision; writing—review and editing; writing—original draft; investigation. **Sarah Jackson:** Funding acquisition; conceptualization; methodology; supervision; writing—review and editing; writing—original draft; investigation.

## DECLARATION OF INTERESTS

All authors have no conflicts of interest to declare.

## Supporting information


**Figure S1.** Smoking prevalence by ethnicity; pooled 2013–2025.
**Figure S2.** Smoking prevalence by gender and ethnicity; pooled 2013–2025.

**Table S1.** Unweighted participant characteristics, %(*n*); pooled 2013–2025.
**Table S2.** Smoking prevalence by ethnicity across regions in England; pooled 2013–2025.
**Table S3.** Smoking prevalence by ethnicity across regions in England; pooled 2013–2025.
**Table S4.** Predicted smoking prevalence by ethnicity and gender; pooled 2013–2025.

## Data Availability

Data are available upon reasonable request. For information on applying for data access, please see https://smokinginengland.info/resources/sts-documents.
