# Supplementary material for: Using aggregated ethnicity categories masks inequalities in smoking prevalence in England
Source: Addiction. 2026 Apr 21;121(8):2234–41. doi: 10.1111/add.70427 (PMC13357640; doi:10.1111/add.70427)
Supplement: Supplementary file 2 — Table S1. Unweighted participant characteristics, %(n); pooled 2013–2025. Table S2. Smoking prevalence by ethnicity across regions in England; pooled 2013–2025. Table S3. Smoking prevalence by ethnicity across regions in England; pooled 2013–2025. Table S4. Predicted smoking prevalence by ethnicity and gender; pooled 2013–2025. [file ADD-121-2234-s001.docx]

| Supplementary Table 1: Unweighted participant characteristics (%(N)): Pooled 2013-2025 | | | | | | | | | | | | | | | | |  |
| --- | --- | --- | --- | --- | --- | --- | --- | --- | --- | --- | --- | --- | --- | --- | --- | --- | --- |
|  | Gender | | Occupational social grade | | | | | Region | | | | | | | | | Age |
|  | Male | Female | AB | C1 | C2 | D | E | North | | | Midlands | | | South | | | M(SD) |
|  |  |  |  |  |  |  |  | East | West | Yorkshire | East | West | East of England | London | East | West |  |
| Asian- Indian | 57.4(3319) | 42.6(2462) | 24.6(1420) | 40.3(2330) | 16.4(945) | 13.2(761) | 5.6(325) | 1.3(76) | 6.3(364) | 5.4(310) | 16.3(942) | 11.6(672) | 6.6(381) | 40.7(2352) | 9.3(539) | 2.5(145) | 40.0  (15.6) |
| Asian- Pakistani | 62.0(3806) | 38.0(2332) | 15.4(945) | 32.4(1989) | 17.9(1096) | 22(1350) | 12.4(758) | 1.3(78) | 15.2(932) | 19.2(1177) | 2.5(156) | 30.7(1881) | 3.3(200) | 21.6(1328) | 5.7(351) | 0.6(35) | 36.8  (14.3) |
| Asian- Bangladeshi | 57.9(1199) | 42.1(873) | 11.1(229) | 34.4(712) | 18.3(379) | 24.4(506) | 11.9(246) | 1.1(23) | 5.4(111) | 3.5(72) | 2.3(47) | 10.5(217) | 4.9(102) | 67.5(1399) | 3.8(78) | 1.1(23) | 34.6  (12.3) |
| Asian- Chinese | 51.3(532) | 48.75(506) | 22.1(229) | 58.8(610) | 9.5(99) | 4.5(47) | 5.1(53) | 1.7(18) | 10.6(110) | 6.7(69) | 5(52) | 18.3(190) | 9.3(96) | 32.3(335) | 11.1(115) | 5.1(53) | 32.3  (13.6) |
| Other Asian ethnicities | 54.7(1062) | 45.3(879) | 20.0(389) | 41(795) | 16(311) | 14.9(289) | 8.1(157) | 1.3(25) | 6.4(125) | 5.0(97) | 4.6(89) | 9.2(179) | 7.2(139) | 50.9(988) | 11.8(228) | 3.7(71) | 37.2  (15.0) |
| Black- African | 49.6(3009) | 50.5(3064) | 18.4(1115) | 39.3(2388) | 16.3(989) | 16(973) | 10(608) | 1.2(75) | 7.3(445) | 4.3(259) | 4.5(274) | 10(607) | 8.2(499) | 53.4(3245) | 8.9(543) | 2.1(126) | 35.4  (14.1) |
| Black- Caribbean | 43.2(1314) | 56.8(1728) | 17.0(517) | 37.5(1142) | 19.7(599) | 11.4(347) | 14.4(437) | 0.2(6) | 3.1(95) | 3.2(96) | 3.9(117) | 11.1(337) | 5.6(170) | 64.8(1970) | 6.1(184) | 2.2(67) | 45.2  (17.9) |
| Other Black ethnicity | 48.5(433) | 51.51(460) | 18.3(163) | 39.6(354) | 17.7(158) | 13.1(117) | 11.3(101) | 0.6(5) | 7.6(68) | 5.9(53) | 4.5(40) | 10.4(93) | 5.9(53) | 53.6(479) | 9.3(83) | 2.1(19) | 38.7  (15.4) |
| White and Black Caribbean | 43.2(579) | 56.8(762) | 18.1(242) | 40.1(538) | 17.1(229) | 12.2(163) | 12.6(169) | 0.8(10) | 9(120) | 9.6(128) | 6.7(90) | 14.3(192) | 8.6(115) | 34.2(459) | 11(148) | 5.9(79) | 35.6  (14.7) |
| White and Black African | 47.4(380) | 52.56(421) | 21.7(174) | 41.7(334) | 14(112) | 12.4(99) | 10.2(82) | 2.8(22) | 14(112) | 5.9(47) | 5(40) | 6.2(50) | 10(80) | 40.3(323) | 11.4(91) | 4.5(36) | 36.1  (14.9) |
| White and Asian | 50.7(612) | 49.3(595) | 28.2(340) | 43.5(525) | 12.3(149) | 8.5(103) | 7.5(90) | 2.1(25) | 10.9(132) | 9.5(115) | 7.6(92) | 10.1(122) | 9.3(112) | 31.4(379) | 13.9(168) | 5.1(62) | 36.4  (15.1) |
| Multiple other ethnicities | 46.3(604) | 53.7(701) | 27.6(360) | 43.8(572) | 12.3(161) | 7.7(101) | 8.5(111) | 1.8(23) | 9.8(128) | 6.1(80) | 5.1(67) | 7.6(99) | 9.5(124) | 39.9(521) | 13.7(179) | 6.4(84) | 37.9  (15.3) |
| White- British | 50.0  (90137) | 50.0  (90232) | 26.0  (46858) | 35.8  (64567) | 18.1  (32696) | 10.0  (18056) | 10.1  (18192) | 6.0  (10743) | 15.2  (27383) | 11.4  (20477) | 9.0  (16136) | 10.2  (18426) | 11.8  (21218) | 9.3  (16791) | 15.9  (28618) | 11.4  (20577) | 52.4  (18.9) |
| White- Irish | 52.4(1272) | 47.6(1155) | 29.5(716) | 36.1(875) | 14.1(341) | 9.2(223) | 11.2(272) | 2.2(53) | 12.9(314) | 6.9(168) | 6.1(147) | 8.5(206) | 10(242) | 32.1(778) | 15.1(367) | 6.3(152) | 54.1  (18.0) |
| White- Gypsy, Traveller | 56.8(109) | 43.23(83) | 14.6(28) | 23.4(45) | 20.8(40) | 22.4(43) | 18.8(36) | 4.2(8) | 10.4(20) | 9.9(19) | 5.7(11) | 13(25) | 15.1(29) | 24(46) | 14.6(28) | 3.1(6) | 39.6  (16.7) |
| White Other | 45.5(5613) | 54.5(6723) | 22.1(2724) | 33.4(4123) | 19.9(2455) | 18.9(2336) | 5.7(698) | 1.3(160) | 6.8(835) | 4.8(595) | 6.4(793) | 7.4(912) | 10(1234) | 43(5305) | 13(1609) | 7.2(893) | 39.4  (14.9) |
| Arab | 61.0(465) | 39.0(297) | 22.3(170) | 36.6(279) | 16.8(128) | 12.2(93) | 12.1(92) | 2.5(19) | 8.7(66) | 8(61) | 4.1(31) | 13.7(104) | 4.5(34) | 41.9(319) | 13.8(105) | 3.0(23) | 35.1  (13.9) |
| Other ethnicity | 52.6(1189) | 47.4(1072) | 22.2(501) | 37(836) | 17.6(397) | 14(317) | 9.3(210) | 1.3(30) | 11.1(251) | 7.1(161) | 6(136) | 8(181) | 11.2(254) | 40.1(906) | 9.1(205) | 6.1(137) | 40.0  (15.8) |

| Supplementary Table 2: Smoking prevalence by ethnicity across regions in England; Pooled 2013-2025 | | | | | | |
| --- | --- | --- | --- | --- | --- | --- |
|  | North | | Midlands | | South | |
|  | N | %(95% CI) | N | %(95% CI) | N | %(95% CI) |
| Asian- Indian | 78 | 11.1(8.8-13.9) | 181 | 8.7(7.4-10.1) | 267 | 9.2(8.1-10.5) |
| Asian- Pakistani | 335 | 15.1(13.5-16.7) | 306 | 13.8(12.3-15.4) | 182 | 11.0(9.5-12.8) |
| Asian- Bangladeshi | 37 | 20.3(14.7-27.2) | 63 | 16.7(12.9-21.4) | 230 | 15.1(13.2-17.2) |
| Asian- Chinese | 28 | 13.4(9.0-19.4) | 38 | 11.2(8.1-15.4) | 56 | 10.2(7.8-13.3) |
| Other Asian ethnicity | 25 | 11.4(7.3-17.3) | 53 | 14.1(10.6-18.4) | 161 | 12.3(10.5-14.4) |
|  |  |  |  |  |  |  |
| Black- African | 66 | 8.3(6.3-10.9) | 91 | 6.2(5-7.7) | 292 | 7.4(6.6-8.4) |
| Black- Caribbean | 33 | 15.8(11.1-22.0) | 88 | 14.4(11.5-17.7) | 403 | 18.2(16.5-20.1) |
| Other Black ethnicity | 15 | 10.9(6.5-17.8) | 33 | 16.7(11.6-23.3) | 101 | 18.8(15.5-22.7) |
|  |  |  |  |  |  |  |
| White and Black Caribbean | 72 | 28.6(22.7-35.2) | 121 | 31.1(26.3-36.3) | 177 | 28.1(24.4-32.1) |
| White and Black African | 37 | 22.8(16.6-30.4) | 35 | 19.4(13.7-26.7) | 88 | 20(16.1-24.5) |
| White and Asian | 67 | 23.6(18.7-29.5) | 53 | 17.3(13.2-22.3) | 122 | 21.2(17.8-25) |
| Multiple other ethnicities | 61 | 26.1(20.4-32.6) | 66 | 24.7(19.6-30.7) | 169 | 22.1(19.1-25.4) |
|  |  |  |  |  |  |  |
| White- British | 11,044 | 19.1(18.8-19.5) | 9,213 | 17.1(16.7-17.4) | 10,451 | 16.4(16.1-16.7) |
| White- Irish | 123 | 23.3(19.6-27.5) | 118 | 20.9(17.5-24.7) | 220 | 18(15.7-20.4) |
| White- Gypsy /Traveller | 17 | 38.1(24.5-53.8) | 23 | 35.7(24.2-49.2) | 33 | 42.5(31.5-54.3) |
| White Other | 443 | 28.4(26.0-30.8) | 698 | 25(23.3-26.8) | 1,855 | 24.6(23.6-25.7) |
|  |  |  |  |  |  |  |
| Arab | 27 | 19.2(13.1-27.1) | 26 | 12.6(8.4-18.4) | 109 | 26.2(21.8-31) |
| Other ethnicity | 89 | 21.0(17.1-25.6) | 97 | 18.2(14.9-22) | 216 | 17.7(15.4-20.1) |
| Due to small cell counts, regions were aggregated into:  North= North East, North West, Yorkshire,  Midlands= East Midlands, West Midlands, East of England  South= London, South East, South West  N are unweighted and %s are weighted | | | | | | |

| Supplementary Table 3: Smoking prevalence by ethnicity across regions in England; Pooled 2013-2025 | | | | |
| --- | --- | --- | --- | --- |
|  | ABC1 | | C2DE | |
|  | N | %(95% CI) | N | %(95% CI) |
|  |  |  |  |  |
| Asian- Indian | 326 | 8.4(7.5-9.4) | 200 | 10.5(9.1-12.1) |
| Asian- Pakistani | 381 | 13(11.7-14.3) | 442 | 13.9(12.6-15.2) |
| Asian- Bangladeshi | 143 | 14.9(12.7-17.5) | 187 | 16.6(14.3-19.2) |
| Asian- Chinese | 93 | 10.0(8.1-12.2) | 29 | 14.8(10.2-21) |
| Other Asian ethnicity | 130 | 10.6(8.9-12.6) | 109 | 14.9(12.2-17.9) |
|  |  |  |  |  |
| Black- African | 253 | 7.0(6.2-7.9) | 196 | 7.5(6.5-8.8) |
| Black- Caribbean | 252 | 14.5(12.8-16.4) | 272 | 19.9(17.6-22.3) |
| Other Black ethnicity | 71 | 14.0(11.1-17.5) | 78 | 20.4(16.3-25.2) |
|  |  |  |  |  |
| White and Black Caribbean | 165 | 21.2(18.2-24.4) | 205 | 37.2(32.9-41.7) |
| White and Black African | 89 | 16.5(13.4-20.1) | 71 | 25.6(20.4-31.6) |
| White and Asian | 144 | 16.7(14.2-19.5) | 98 | 28.0(23.2-33.4) |
| Multiple other ethnicities | 191 | 20.2(17.6-23.1) | 105 | 28.9(24.2-34.0) |
|  |  |  |  |  |
| White- British | 13,743 | 12.2(12.0-12.4) | 16,965 | 24.3(23.9-24.6) |
| White- Irish | 242 | 15.0(13.3-17.0) | 219 | 27.5(24.2-31.0) |
| White- Gypsy /Traveller | 25 | 34.6(23.9-47.0) | 48 | 41.2(32.2-50.9) |
| White Other | 1,237 | 17.7(16.8-18.7) | 1,759 | 32.3(31.0-33.6) |
|  |  |  |  |  |
| Arab | 80 | 17.6(14.2-21.6) | 82 | 26.3(21.3-32.1) |
| Other ethnicity | 188 | 13.5(11.7-15.5) | 214 | 23.7(20.8-26.8) |
| N are unweighted and %s are weighted | | | | |

| Supplementary Table 4: Predicted smoking prevalence by ethnicity and gender; pooled 2013- 2025 | | | | | | |
| --- | --- | --- | --- | --- | --- | --- |
|  | Model 1 | | Model 2 | | Model 3 | |
|  | AOR(95% CI) | p | AOR(95% CI) | p | AOR(95% CI) | p |
| Aggregated Asian, British Asian |  |  |  |  |  |  |
| Asian- Indian | 1.00 | Ref | 1 | Ref | 1.00 | Ref |
| Asian- Pakistani | 1.49(1.31-1.69) | **<.001** | 1.40(1.23-1.59) | **<.001** | 1.22(1.07-1.39) | **.002** |
| Asian- Bangladeshi | 1.81(1.54-2.13) | **<.001** | 1.66(1.41-1.96) | **<.001** | 1.44(1.22-1.71) | **<.001** |
| Asian- Chinese | 1.22(0.97-1.53) | .089 | 1.09(0.87-1.37) | .465 | 1.25(0.99-1.57) | .064 |
| Other Asian ethnicity | 1.41(1.18-1.69) | **<.001** | 1.35(1.13-1.62) | **.001** | 1.29(1.07-1.55) | **.007** |
|  |  |  |  |  |  |  |
| Aggregated Black, Black British |  |  |  |  |  |  |
| Black- African | 1.00 | Ref | 1 | Ref | 1.00 | Ref |
| Black- Caribbean | 2.65(2.28-3.07) | **<.001** | 3.27(2.81-3.80) | **<.001** | 3.34(2.87-3.89) | **<.001** |
| Other Black ethnicity | 2.69(2.16-3.35) | **<.001** | 2.89(2.31-3.63) | **<.001** | 2.92(2.32-3.69) | **<.001** |
|  |  |  |  |  |  |  |
| Aggregated Mixed or Multiple ethnicities |  |  |  |  |  |  |
| White and Black Caribbean | 1.00 | Ref | 1 | Ref | 1.00 | Ref |
| White and Black African | 0.63(0.50-0.79) | **<.001** | 0.62(0.49-0.78) | **<.001** | 0.64(0.50-0.81) | **<001** |
| White and Asian | 0.64(0.52-0.78) | **<.001** | 0.63(0.51-0.78) | **<.001** | 0.70(0.57-0.87) | **.001** |
| Multiple other ethnicities | 0.76(0.62-0.92) | **.005** | 0.78(0.64-0.95) | **.015** | 0.86(0.70-1.06) | .150 |
|  |  |  |  |  |  |  |
| Aggregated White, White British |  |  |  |  |  |  |
| White- British | 1.00 | Ref | 1 | Ref | 1.00 | Ref |
| White- Irish | 1.19(1.06-1.33) | **.003** | 1.24(1.11-1.39) | **<.001** | 1.32(1.18-1.48) | **<.001** |
| White- Gypsy /Traveller | 3.07(2.25-4.21) | **<.001** | 2.34(1.71-3.21) | **<.001** | 1.99(1.43-2.76) | **<.001** |
| Other White | 1.60(1.52-1.67) | **<.001** | 1.26(1.20-1.32) | **<.001** | 1.19(1.13-1.25) | **<.001** |
| AOR=Adjusted odds ratio; CI=Confidence interval.  Model 1: Adjusted by survey year and survey mode  Model 2: Adjusted by age, gender survey year and survey mode  Model 3: Adjusted by socioeconomic status, region, age, gender survey year and survey mode | | | | | | |
