# Supplementary material for: Using aggregated ethnicity categories masks inequalities in smoking prevalence in England
Source: Addiction. 2026 Apr 21;121(8):2234–41. doi: 10.1111/add.70427 (PMC13357640; doi:10.1111/add.70427)

Figure S1: Smoking prevelence by ethnicity; pooled 2013-2025

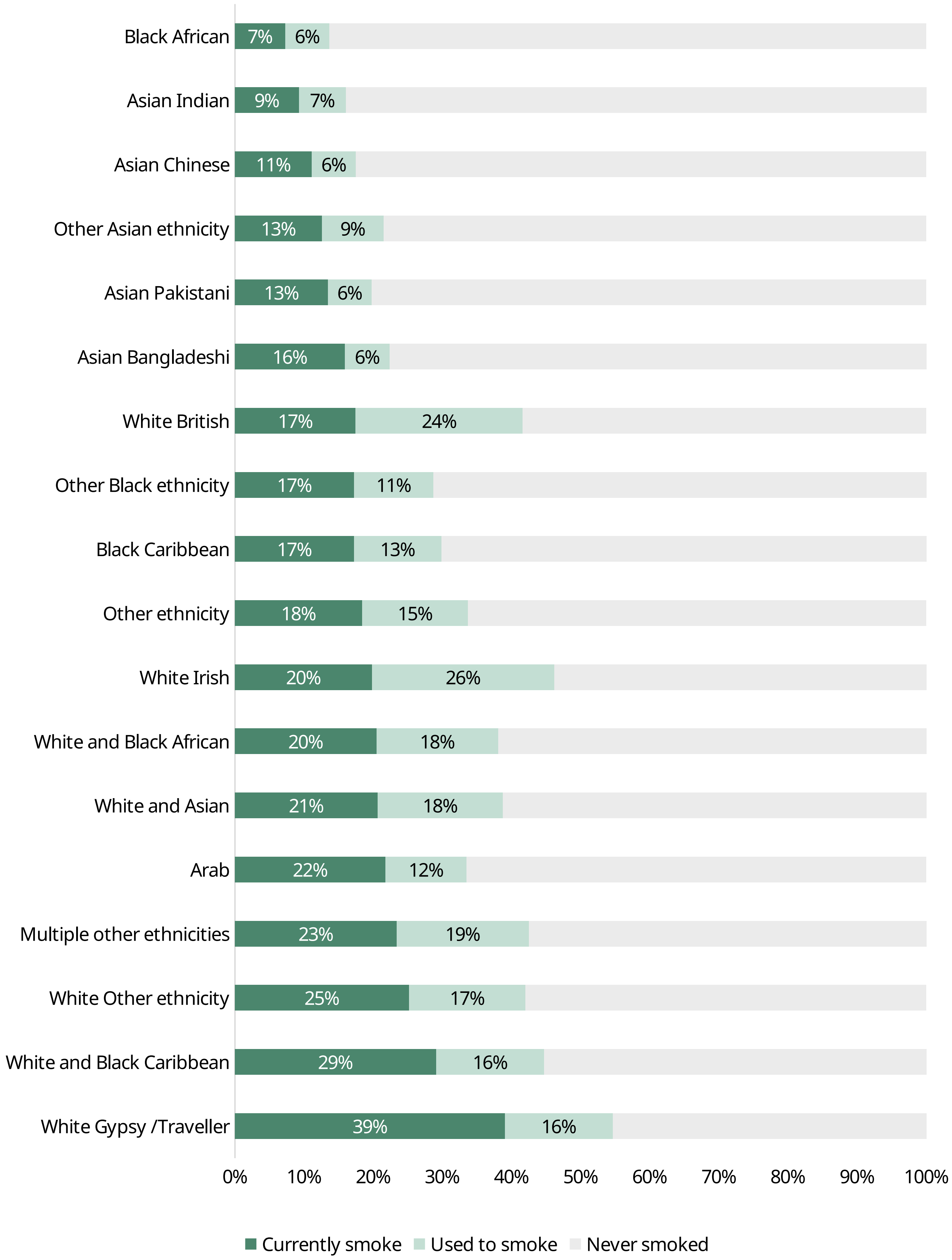

Figure S2: Smoking prevelence by gender and ethnicity; pooled 2013-2025

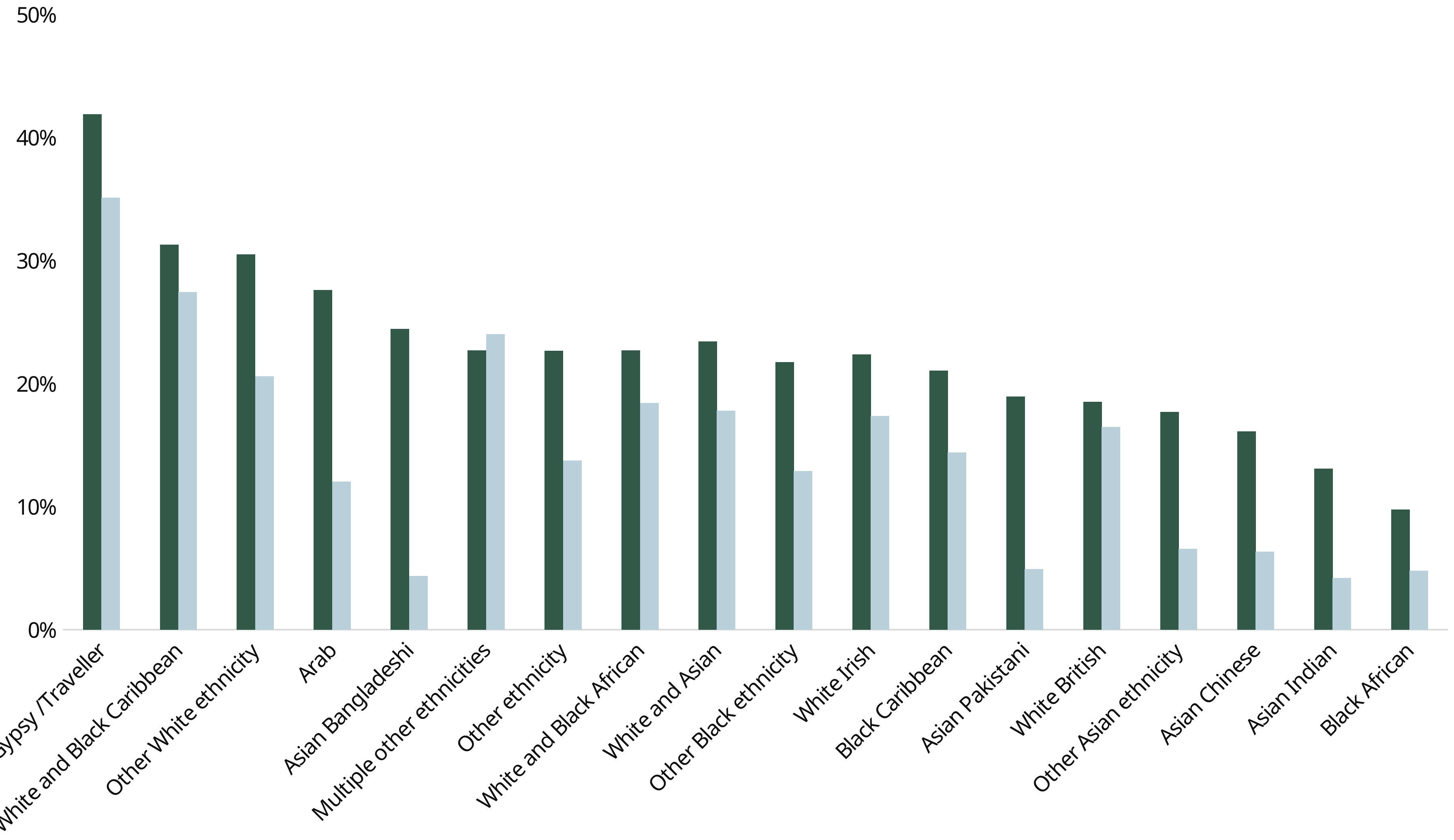

Supplement: Supplementary file 2 [file ADD-121-2234-s002.docx]
